# Immigrant IBD Patients in Spain Are Younger, Have More Extraintestinal Manifestations and Use More Biologics Than Native Patients

**DOI:** 10.3389/fmed.2022.823900

**Published:** 2022-02-01

**Authors:** Ana Gutiérrez, Pedro Zapater, Elena Ricart, María González-Vivó, Jordi Gordillo, David Olivares, Isabel Vera, Míriam Mañosa, Javier P. Gisbert, Mariam Aguas, Eugenia Sánchez-Rodríguez, Maia Bosca-Watts, Viviana Laredo, Blau Camps, Ignacio Marín-Jiménez, Yamile Zabana, María Dolores Martín-Arranz, Roser Muñoz, Mercè Navarro, Eva Sierra, Lucía Madero, Milagros Vela, José Lázaro Pérez-Calle, Empar Sainz, Xavier Calvet, Lara Arias, Victor Morales, Fernando Bermejo, Luis Fernández-Salazar, Manuel Van Domselaar, Luisa De Castro, Cristina Rodríguez, Carmen Muñoz-Villafranca, Rufo Lorente, Montserrat Rivero, Eva Iglesias, Belén Herreros, David Busquets, Joan Riera, María Pilar Martínez-Montiel, Marta Roldón, Oscar Roncero, Esther Hinojosa, Mónica Sierra, Jesús Barrio, Ruth De Francisco, José Huguet, Olga Merino, Daniel Carpio, Daniel Ginard, Fernando Muñoz, Marta Piqueras, Pedro Almela, Federico Argüelles-Arias, Guillermo Alcaín, Luis Bujanda, Noemí Manceñido, Alfredo J. Lucendo, Pilar Varela, Iago Rodríguez-Lago, Laura Ramos, Laura Sempere, Eva Sesé, Manuel Barreiro-de Acosta, Eugeni Domènech, Rubén Francés

**Affiliations:** ^1^Servicio Medicina Digestiva, Hospital General Universitario Alicante, Alicante, Spain; ^2^IIS Isabial, Hospital General Universitario Alicante, Alicante, Spain; ^3^Centro de Investigación Biomédica en Red de Enfermedades Hepáticas y Digestivas (CIBERehd), Instituto de Salud Carlos III, Madrid, Spain; ^4^Unidad Farmacología Clínica, Hospital General Universitario Alicante, Alicante, Spain; ^5^Instituto IDIBE, Universidad Miguel Hernández, San Juan de Alicante, Spain; ^6^Servicio de Medicina Digestiva Hospital Clínic, Instituto de Investigaciones Biomédicas August Pi i Sunyer (IDIBAPS), Barcelona, Spain; ^7^Servicio Medicina Digestiva, Hospital del Mar, IMIM (Hospital del Mar Medical Research Institute), Barcelona, Spain; ^8^Servicio Patología Digestiva, Hospital de la Santa Creu I Sant Pau, Barcelona, Spain; ^9^Servicio Medicina Digestiva, Hospital Universitario Clínico San Carlos, Madrid, Spain; ^10^Servicio Aparato Digestivo, Hospital Universitario Puerta de Hierro, Madrid, Spain; ^11^Servicio Aparato Digestivo, Hospital Universitari Germans Trias I Pujol, Badalona, Spain; ^12^Servicio de Aparato Digestivo, Hospital Universitario de La Princesa, Instituto de Investigación Sanitaria Princesa (IIS-IP), Universidad Autónoma de Madrid (UAM), Madrid, Spain; ^13^Servicio Medicina Digestiva, Hospital Universitario La Fé, Valencia, Spain; ^14^Servicio Medicina Digestiva, Hospital Universitario Ramón y Cajal, Madrid, Spain; ^15^Servicio Medicina Digestiva, Hospital Clínico Universitario de Valencia, Valencia, Spain; ^16^Servicio Medicina Digestiva, Hospital Clinico Universitario Lozano Blesa, Zaragoza, Spain; ^17^Servicio Medicina Digestiva, Hospital Universitario de Bellvitge, Barcelona, Spain; ^18^Servicio Medicina Digestiva, Hospital Gregorio Marañón, Madrid, Spain; ^19^Gastroenterology Department, Instituto de Investigación Biomédica Gregorio Marañón IiSGM, Madrid, Spain; ^20^Servicio Medicina Digestiva, Hospital Universitari Mútua Terrassa, Terrassa, Spain; ^21^Servicio Medicina Digestiva, Hospital La Paz, Madrid, Spain; ^22^Servicio Medicina Digestiva, Hospital de Sant Joan Despí Moisès Broggi, Barcelona, Spain; ^23^Servicio Medicina Digestiva, Hospital Universitario Miguel Servert, Zaragoza, Spain; ^24^Servicio Medicina Digestiva, Hospital General Universitario de Elche, Elche, Spain; ^25^Servicio Medicina Digestiva, Hospital Nuestra Señora de la Candelaria, Santa Cruz de Tenerife, Spain; ^26^Servicio Medicina Digestiva, Hospital Universitario Fundación de Alcorcón, Madrid, Spain; ^27^Servicio Medicina Digestiva, Hospital Sant Joan de Déu - Althaia, Manresa, Spain; ^28^Unitat Malalties Digestives, Hospital de Sabadell, Institut Universitari Parc Tauli, Universidad Autónoma de Barcelona (UAB), Barcelona, Spain; ^29^Servicio Medicina Digestiva, Hospital Universitario de Burgos, Burgos, Spain; ^30^Servicio Medicina Digestiva, Hospital General de Granollers, Barcelona, Spain; ^31^Servicio Medicina Digestiva, Hospital de Fuenlabrada, Fuenlabrada, Spain; ^32^IIS Hospital La Paz IdiPaz-Madrid, Madrid, Spain; ^33^Servicio Medicina Digestiva, Hospital Clínico de Valladolid, Madrid, Spain; ^34^Servicio Medicina Digestiva, Hospital de Torrejón de Ardoz, Madrid, Spain; ^35^Department of Gastroenterology, Xerencia Xestion Integrada de Vigo- SERGAS. IIS Galicia Sur. SERGAS-UVIG, Vigo, Spain; ^36^Servicio Medicina Digestiva, Complejo Hospitalario de Navarra, Pamplona, Spain; ^37^Servicio Medicina Digestiva, Hospital Universitario de Basurto, Bilbao, Spain; ^38^Servicio Medicina Digestiva, Hospital General Ciudad Real, Ciudad Real, Spain; ^39^Servicio Medicina Digestiva, Hospital Universitario Marqués de Valdecilla and IDIVAL, Santander, Spain; ^40^Servicio Medicina Digestiva, Hospital Universitario Reina Sofía, Córdoba, Spain; ^41^Instituto Maimónides de Investigación Biomédica de Córdoba (IMIBIC), Córdoba, Spain; ^42^Servicio Medicina Digestiva, Hospital Marina Baixa, Villajoyosa, Spain; ^43^Servicio Medicina Digestiva, Hospital de Girona Dr. Trueta/ICO, Girona, Spain; ^44^Servicio Medicina Digestiva, Hospital Universitario Son LLàtzer, Palma de Mallorca, Spain; ^45^Servicio Medicina Digestiva, Hospital 12 de Octubre, Madrid, Spain; ^46^Servicio Cirugía General y del Aparato Digestivo, Hospital San Jorge, Huesca, Spain; ^47^Servicio Medicina Digestiva, Hospital General La Mancha Centro, Ciudad Real, Spain; ^48^Servicio Medicina Digestiva, Hospital de Manises, Valencia, Spain; ^49^Servicio Medicina Digestiva, Complejo Asistencial Universitario de León, León, Spain; ^50^Hospital Universitario Rio Hortega, Valladolid, Spain; ^51^Hospital Universitario Central de Asturias and ISPA, Oviedo, Spain; ^52^Consorcio Hospital General Universitario de Valencia, Valencia, Spain; ^53^Servicio Medicina Digestiva, Hospital de Cruces, Bilbao, Spain; ^54^Complejo Hospitalario Universitario Pontevedra, Pontevedra, Spain; ^55^Servicio Medicina Digestiva, Hospital Universitario Son Espases, Palma de Mallorca, Spain; ^56^Servicio Medicina Digestiva, Hospital Clínico Universitario Salamanca, Salamanca, Spain; ^57^Servicio Medicina Digestiva, Consorci Sanitari Terrasa, Barcelona, Spain; ^58^Servicio Medicina Digestiva, Hospital General Universitario Castellón, Castellón de la Plana, Spain; ^59^Servicio Medicina Digestiva, Hospital Universitario Virgen Macarena, Sevilla, Spain; ^60^Servicio Medicina Digestiva, Hospital Clínico Virgen de la Victoria, Málaga, Spain; ^61^Servicio Medicina Digestiva, Hospital Universitario Donostia, San Sebastián, Spain; ^62^Instituto Biodonostia, Universidad Pais Vasco, San Sebastián, Spain; ^63^Servicio Medicina Digestiva, Hospital Infanta Sofía, Madrid, Spain; ^64^Servicio Medicina Digestiva, Hospital General Tomelloso, Ciudad Real, Spain; ^65^Servicio Medicina Digestiva, Hospital Cabueñes, Gijón, Spain; ^66^Servicio de Aparato Digestivo, Hospital Universitario de Galdakao, IIS Biocruces, Galdakao, Spain; ^67^Facultad de Medicina, University of Deusto, Bilbao, Spain; ^68^Servicio Medicina Digestiva, Hospital Universitario La Laguna, Santa Cruz Tenerife, Spain; ^69^Servicio Medicina Digestiva, Hospital Arnau de Vilanova, Lleida, Spain; ^70^Servicio Aparato Digestivo, Hospital Universitario Santiago, Santiago de Compostela, Spain; ^71^Hepatic and Intestinal Immunobiology Group, Dpto. Medicina Clínica, Universidad Miguel Hernández, San Juan de Alicante, Spain; ^72^Instituto de Investigación, Desarrollo e Innovación en Biotecnología Sanitaria de Elche (IDiBE), Universidad Miguel Hernández, Elche, Spain

**Keywords:** immigrant, phenotype, biologics, inflammatory bowel disease, Crohn's disease, ulcerative colitis

## Abstract

**Background:**

Previous studies comparing immigrant ethnic groups and native patients with IBD have yielded clinical and phenotypic differences. To date, no study has focused on the immigrant IBD population in Spain.

**Methods:**

Prospective, observational, multicenter study comparing cohorts of IBD patients from ENEIDA-registry who were born outside Spain with a cohort of native patients.

**Results:**

We included 13,524 patients (1,864 immigrant and 11,660 native). The immigrants were younger (45 ± 12 vs. 54 ± 16 years, *p* < 0.001), had been diagnosed younger (31 ± 12 vs. 36 ± 15 years, *p* < 0.001), and had a shorter disease duration (14 ± 7 vs. 18 ± 8 years, *p* < 0.001) than native patients. Family history of IBD (9 vs. 14%, *p* < 0.001) and smoking (30 vs. 40%, *p* < 0.001) were more frequent among native patients. The most prevalent ethnic groups among immigrants were Caucasian (41.5%), followed by Latin American (30.8%), Arab (18.3%), and Asian (6.7%). Extraintestinal manifestations, mainly musculoskeletal affections, were more frequent in immigrants (19 vs. 11%, *p* < 0.001). Use of biologics, mainly anti-TNF, was greater in immigrants (36 vs. 29%, *p* < 0.001). The risk of having extraintestinal manifestations [OR: 2.23 (1.92–2.58, *p* < 0.001)] and using biologics [OR: 1.13 (1.0–1.26, *p* = 0.042)] was independently associated with immigrant status in the multivariate analyses.

**Conclusions:**

Compared with native-born patients, first-generation-immigrant IBD patients in Spain were younger at disease onset and showed an increased risk of having extraintestinal manifestations and using biologics. Our study suggests a featured phenotype of immigrant IBD patients in Spain, and constitutes a new landmark in the epidemiological characterization of immigrant IBD populations in Southern Europe.

## HIGHLIGHTS

What is known?

- Studies evaluating phenotypic characteristics of immigrant IBD populations show specific features that are not always reproduced in native patients.
- Few studies have evaluated the characteristics of immigrant IBD patients in Southern Europe and, to date, none has focused on the immigrant IBD population in Spain.

What is new here?

- The main ethnic groups of immigrant IBD patients in Spain are Caucasian, Latin American and Arab.
- Immigrant IBD patients in Spain are younger and have more extraintestinal manifestations than native-born patients. Accordingly, the use of biologics is more frequent among immigrants.
- The study represents a new landmark in the epidemiologic characterization of immigrant patients with IBD in Southern Europe.

## Introduction

Inflammatory bowel disease (IBD) is a term that covers Crohn's disease (CD) and ulcerative colitis (UC), which are chronic, progressive and disabling diseases characterized by cycles of remission and relapse. Though the cause of IBD is not yet fully understood, its pathogenesis has been linked to complex interactions between genetic, epithelial, immune, microbial and environmental factors (1, 2). Although more than 240 IBD-associated risk variants have been identified to date by large genome-wide association studies (3), genetic background seems only to partially explain susceptibility to the disease and in most cases, environmental risk factors are also involved in disease onset and progression (4, 5). However, studies focusing on causative environmental factors are challenging and have produced conflicting results (6).

The epidemiology of IBD has changed over time and is influenced by geographical variations, suggesting that environmental factors play a role in modifying its expression (7). In fact, newer epidemiologic studies define IBD as a global disease, suggesting that its incidence either continues to grow steadily or remains stable in western countries while rapidly increasing in developing countries (8), possibly owing to their increasingly westernized lifestyle (9). Moreover, studies carried out among migrants show that they tend to acquire the disease incidence of their adopted country rather than the country of origin, reflecting the importance of the environmental risk factors such as lifestyle, pollution and diet (10–12).

Migration has increased in the past decades together with globalization. Several studies have been conducted in large, well-defined migrant groups such as Mexicans living in the USA and people from India living in Europe, the USA or the Middle East (13). A recent review on the epidemiology of IBD in migrant racial and ethnic groups revealed differences in disease incidence, prevalence and phenotypes between immigrants and native patients in individual studies (14). However, few studies of this kind have analyzed European populations. Although there is evidence showing an increased risk of IBD in Spanish people who have migrated to Western Europe (15), to date no study has explored the characteristics of immigrant IBD patients in Spain, which has a population of 47 million people and more than 5 million foreign residents (www.ine.es/en).

This study aimed to explore the characteristics of immigrant IBD patients in Spain compared with native IBD patients, focusing on potential differences in age of disease onset, IBD phenotype, and therapeutic requirements.

## Methods

### Study Design

This was an observational, prospective, multicenter, nationwide study carried out with data from ENEIDA project (16). ENEIDA is a large, prospectively maintained nationwide registry of IBD patients in Spain. It was set up in 2006 by the Spanish Working Group on Crohn's Disease and Ulcerative Colitis (GETECCU) and undergoes continuous external monitoring to ensure completeness and consistency of the entered data. At the time of the study, the database contained data from over 69,000 patients from ~100 actively participating centers.

### Study Population and Data Collection

The immigrant cohort of our study comprised all adult IBD patients born outside of Spain (first-generation immigrants) and included in the ENEIDA registry. Any conflicting data regarding date of migration to Spain, country of birth, ethnicity, or date of IBD diagnosis were checked against patient's clinical records by ENEIDA project investigators. The second cohort was a large, randomly selected sample of adult native IBD patients included in the ENEIDA database. Native IBD patients were born in Spain and were all Caucasians. Inclusion criteria were diagnosis with UC, CD or IBD unclassified (IBDU) based on the accepted criteria of the European Crohn's and Colitis Organization (ECCO) (17); diagnosis before 2015, to ensure follow-up of at least 5 years; and age 16 years or older. The ENEIDA project was approved by research ethics committees in all participating centers and the GETECCU scientific committee. All patients gave written informed consent before being enrolled in the ENEIDA registry.

### Patient Demographics, Disease Phenotype and Disease Course

We collected the following demographic characteristics of the included patients: age, sex, smoking status, ethnicity, country of birth, date of migration to Spain, date of IBD diagnosis, duration of the disease, and family history of IBD. The ethnic groups were classified as Caucasian, Latin American, Arab, Asian, African, Roma, and Jewish. Using the United Nations Standard Country or Area Codes for Statistical Use, M49 standard (https://unstats.un.org/unsd/methodology/m49/) we categorized the birthplace of immigrants into the following geographical regions: Northern Africa, Central Africa, South America, Northern America, Central America, Caribbean, Eastern Europe, Western Europe, Southern Europe, Northern Europe, Southern Asia, Western Asia, Eastern Asia, Southeast Asia, and Oceania.

We used the Montreal classification of IBD to classify disease phenotypes according to extent of UC and location and behavior of CD (18). Surgical procedures were defined as interventions related directly to IBD or its complications, including both abdominal and perianal surgeries. Pharmacological treatment collected included type and time to initiation of first biologic agent and use of immunomodulator agents (methotrexate, azathioprine, or 6-mercaptopurine).

### Statistical Analysis

Continuous variables are reported as means with standard deviations and categorical variables as absolute and relative frequencies. Differences between immigrant and native populations were analyzed using the two-tailed *t* test and Mann-Whitney U test for normally and non-normally distributed quantitative variables, respectively. We analyzed categorical variables using the Chi-square test. Univariate and multivariate logistic regression analyses were performed to assess potential risk factors for the development of EIMs, use of biologics, surgery, and death. Only significant variables from the univariable analysis were entered into the regression model. Estimations of crude odds ratios (ORs) and their 95% confidence intervals (CIs) were estimated using logistic regression analysis. Timeframe between study entry and surgery or initiation of treatment with biologics was analyzed using Kaplan-Meier life-table analysis, as precise dates were known. Log-rank test was used to compare survival curves. All reported *P*-values are 2-sided, and *P*-values lower than 0.05 are considered to indicate statistical significance. All analyses were carried out in the R software environment (Core Team 2014, Vienna, Austria).

## Results

### Demographic Characteristics

A total of 13,524 patients were included in this study, 1,864 of whom were immigrants born outside Spain. The comparative cohort included 11,660 Caucasian IBD patients born in Spain. Table 1 summarizes the main demographic characteristics of immigrants and native patients. Median age of the immigrant group at the time of arrival in Spain was 26 years (range 0–78 years). No differences in IBD diagnosis and sex were observed between the two groups comprising all IBD patients, but more native than immigrant patients diagnosed with UC were men. Immigrant IBD patients were younger at inclusion, were diagnosed earlier in life, and had a shorter disease duration than native patients. These differences persisted when CD and UC patients were analyzed separately (Supplementary Tables 1, 2). Immigrants aged younger than 15 years on arrival in Spain were significantly younger at disease onset than those who migrated after age 15 (23.24 ± 11.34 vs. 32.12 ± 11.28, *p* < 0.001, Supplementary Table 3). Family history of IBD and smoking (present or past) were more frequent in native patients than in immigrants (14.3 vs. 9.4%, *p* = 0.001; and 40.6 vs. 28%, *p* = 0.001, respectively). No differences were found regarding the frequency of appendectomies between the two groups. Seventy-one percent of immigrant patients were diagnosed with IBD in Spain. Supplementary Table 4 compares the main demographic characteristics of immigrants diagnosed in Spain vs. outside Spain.

**Table 1:** Demographic and clinical characteristics of the immigrant and native-born IBD population in Spain.

	**Immigrants**	**Natives**	***P*** **-value**
	**(***n*** = 1,864)**	**(***n*** = 11,660)**	
Gender, female (n,%)	936 (50.2)	5444 (46.7)	0.005
Mean current age (SD)	45.3 (12.6)	54.5 (16.0)	<0.001
Mean age at IBD diagnosis	31.2 (12.0)	36.5 (15.6)	<0.001
Mean duration of disease (SD)	14.0 (7.2)	18.0 (8.7)	<0.001
**Smoking habit**			
no (*n*,%)	1181 (69.3)	6952 (59.5)	<0.001
yes (*n*,%)	296 (17.4)	2227 (19.1)	
Ex (*n*,%)	227 (13.3)	2508 (21.5)	
Crohn's disease (*n*,%)	777 (41.8)	4755 (40.8)	0.068
Ulcerative colitis (*n*,%)	1029 (55.4)	6665 (57.2)	
Unclassified IBD (*n*,%)	52 (2.8)	240 (2.1)	
**Crohn's age at diagnosis**			
A1 (<16y) (n,%)	67 (8.6)	275 (5.8)	<0.001
A2 (16-40y) (*n*,%)	586 (75.3)	3223 (67.8)	
A3 (>40y) (*n*,%)	125 (16.1)	1257 (26.4)	
**Crohn's location**			
L1 (ileal) (*n*,%)	154 (28.5)	1025 (28.4)	0.368
L2 (colonic) (*n*,%)	112 (20.7)	640 (17.7)	
L3 (ileocolonic) (*n*,%)	233 (43.1)	1636 (45.3)	
L4 (upper GI) (*n*,%)	42 (7.8)	308 (8.5)	
**Crohn's behavior**			
B1 (inflammatory)	482 (63.9)	2744 (57.7)	0.002
B2 (stricturing) (*n*,%)	144 (19.1)	1158 (24.4)	
B3 (perforating) (*n*,%)	128 (17.0)	853 (17.9)	
Perianal perforating (*n*;%)	242 (13.7)	1776 (15.4)	0.069
**Ulcerative colitis extent**			
Proctitis (*n*,%)	186 (29.7)	1478 (31.9)	0.031
Left sided colitis (*n*,%)	357 (57.0)	2689 (58.1)	
Extensive colitis (*n*,%)	83 (13.3)	459 (9.9)	
Extraintestinal manifestations (*n*;%)	318 (18.9)	1275 (10.9)	<0.001
Family history of IBD (*n*,%)	155 (9.4)	1676 (14.3)	<0.001

**Figure 1:**
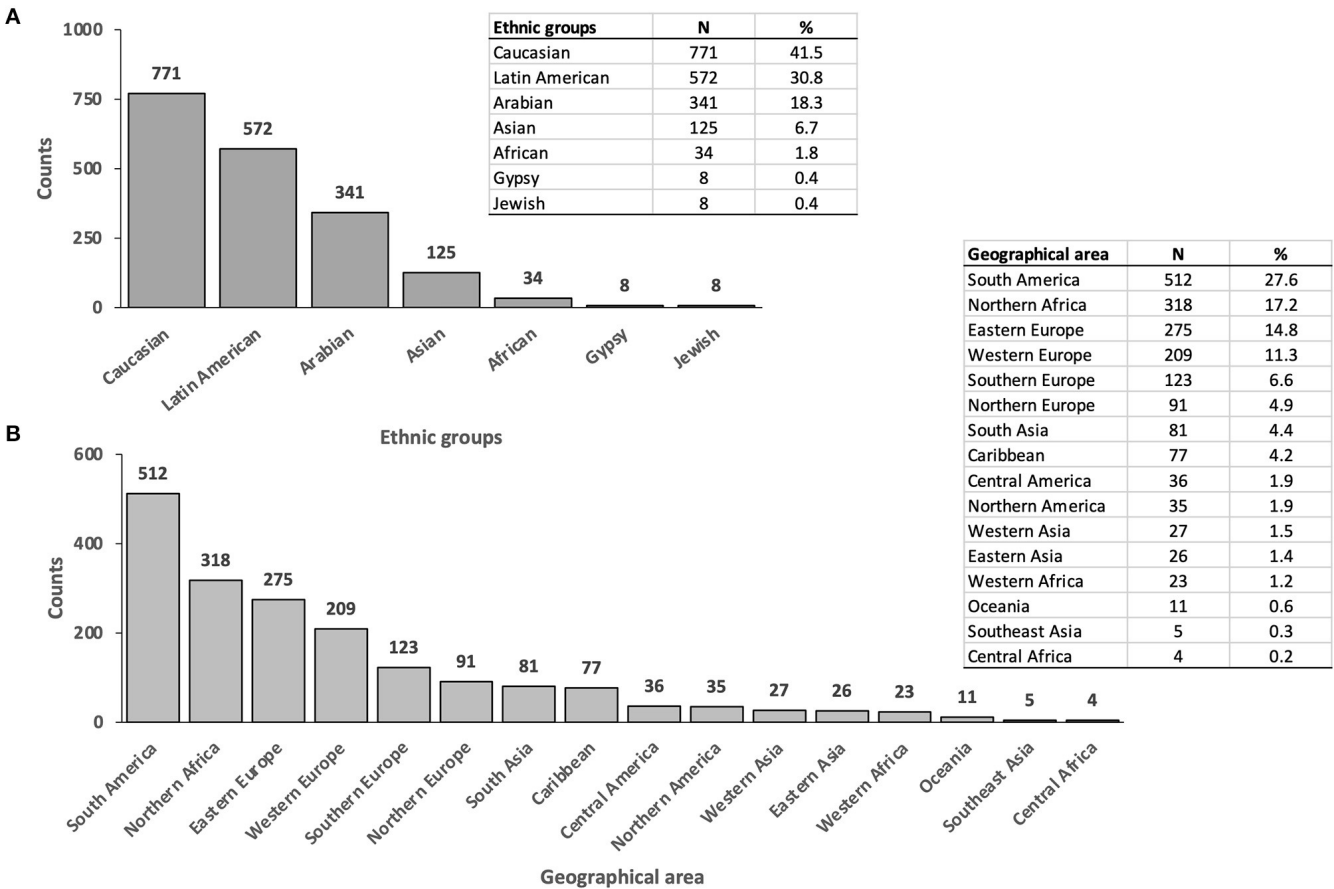
**(A)** Distribution of immigrant population by ethnicity. **(B)** Distribution of immigrant population by geographical area of birth according to OMS Standard Country or Area Codes for Statistical Use, M49 standard.

Figure 1A shows the distribution by ethnicity of the immigrant cohort. The most prevalent ethnic group was Caucasian (41.5%, 771), followed by Latin American (30.8%, 572) and Arab (18.3%, 341), whereas Asians represented a minority (6.7%, 125). Supplementary Table 5 shows the characteristics of immigrant patients by ethnicity. UC diagnosis was more common among Latin American and Asian IBD patients compared with Caucasians and Arabs. The distribution of the immigrant population by geographical area of birth is represented in Figure 1B. South America was the most frequent birthplace, followed by North Africa and Eastern Europe.

### Disease Phenotype

Phenotypic IBD characteristics of both cohorts are displayed in Table 1. Disease location in CD patients did not differ between the two groups. Regarding the age of disease onset, the number of patients diagnosed under 16 years (A1) was significantly higher among immigrant compared with native CD patients. In contrast, the number of patients diagnosed over 40 years (A3) was significantly higher in the native group. Stricturing behavior was more frequent among native CD patients, although the multivariate analysis found no independent association between stricturing behavior and being native. Similarly, stricturing behavior was not independently associated with smoking but was associated with longer disease duration (Supplementary Table 6). We found no significant difference in the prevalence of perianal disease between the two groups. In UC patients, the distribution of ulcerative proctitis, left sided UC and extensive UC was similar in immigrant and native patients.

**Figure 2:**
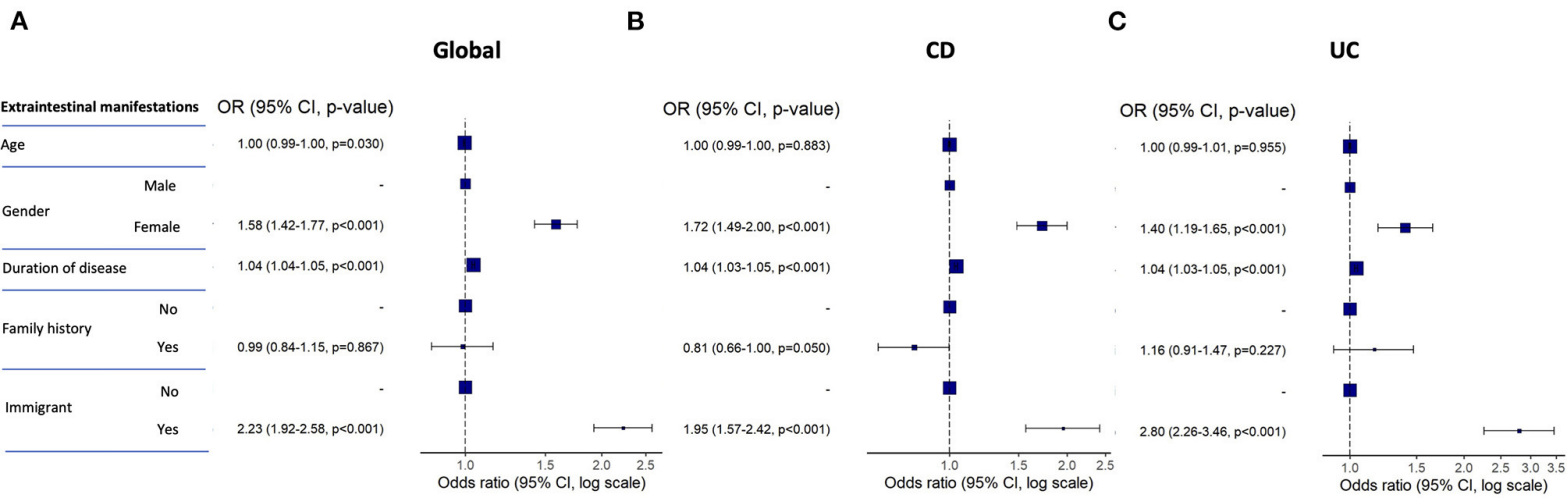
Forest plots representing the odds ratio for presenting with EIMs in the multivariate analysis for the global **(A)**, CD **(B)**, and UC **(C)** cohorts of patients. CD, Crohn's disease; UC, ulcerative colitis.

EIMs were significantly more frequent among immigrant compared with native IBD patients (Table 1). The list of EIMs is detailed in Table 2. Musculoskeletal affections were the most frequent EIM among patients in both groups. Primary sclerosing cholangitis was more frequent in immigrant compared with native patients only in the cohort of UC patients. In the multivariate analysis, the risk of suffering EIMs among the complete series of IBD patients was independently associated with female sex, longer duration of disease and being an immigrant, while family history of IBD was unrelated (Figure 2A). These factors remained independently associated with an increased risk of EIMs when the patients were divided into CD and UC patients (Figures 2B,C). Supplementary Table 7 describes the complete univariate and multivariate analyses for EIMs in IBD patients.

**Table 2:** Extraintestinal manifestations in the immigrant and native-born IBD population in Spain.

	**Immigrants**	**Natives**	***p*** **-value**
***N*** **(%)**	**(***n*** = 395)**	**(***n*** = 1,608)**	
Peripheral arthritis	164 (42.3)	582 (25.3)	<0.001
Ankylosing spondylitis	52 (14.2)	158 (6.1)	<0.001
Isolated sacroiliitis	29 (13.9)	166 (18.2)	0.176
Erythema nodosum	40 (17.6)	221 (10.5)	0.002
Pyoderma gangrenosum	11 (6.0)	67 (3.2)	0.078
Aphtous stomatitis	31 (8.8)	156 (6.0)	0.005
Uveitis	25 (12.2)	76 (3.8)	<0.001
Episcleritis	6 (3.5)	64 (3.2)	1.000
Primary sclerosing cholangitis	21 (5.9)	41 (1.6)	<0.001
Thrombosis	16 (4.5)	77 (3.0)	0.167

### Therapeutic Requirements and Disease Course

**Figure 3:**
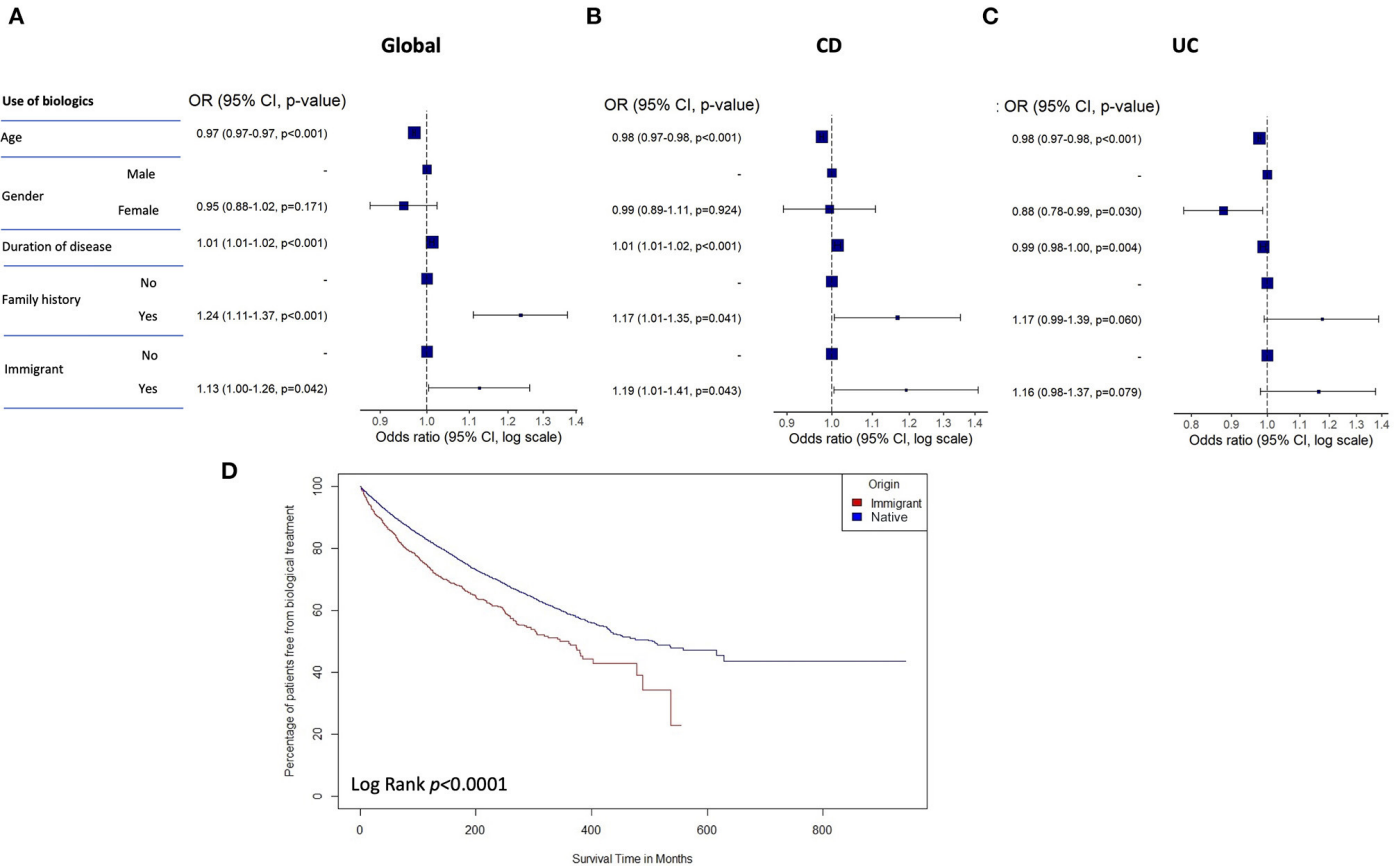
Forest plots representing the odds ratio for using biologics in the multivariate analysis for the global **(A)**, CD **(B)**, and UC **(C)** cohorts of patients. **(D)** Survival analysis of the time to first biologic drug in immigrant vs. native patients. CD, Crohn's disease; UC, ulcerative colitis.

Table 3 summarizes the different medical treatments and surgical procedures in both groups of patients. Endoscopic treatment was more frequent among native patients, while no significant differences between groups were present regarding the use of immunosuppressants (mainly thiopurines). In contrast, the use of biologics was significantly more frequent in the subgroup of immigrants compared with native patients. Of the different biologics, anti-TNF was the most frequently prescribed in both subgroups. Younger age, longer duration of disease, family history of IBD and being a immigrant were also independently associated with the use of biological therapy in the complete series of IBD patients in the multivariate analysis (Figure 3A). While all these variables remained independently associated with the use of biologics in the cohort of CD patients, family history and being a immigrant were not independently associated with an increased risk of using biologics in the multivariate analysis among UC patients (Figures 3B,C). Supplementary Table 8 describes the univariate and multivariate analyses for the use of biologics in IBD patients. Time to first biologic drug during follow-up was significantly earlier in immigrant vs. native patients according to the performed survival analysis of the time to first biologic drug (Figure 3D).

**Table 3:** Medical treatments and surgical procedures in the immigrant and native-born IBD population in Spain.

	**Immigrants**	**Natives**	***p*** **-value**
*N* (%)			
Endoscopic treatment	24/1517 (1.6)	425/10663 (3.9)	<0.001
Immunosuppresants	1006/1835 (54.8)	6126/11651 (52.6)	0.082
Biologics	643/1766 (36.4)	3469/11651 (29.8)	<0.001
AntiTNF	637 (34.2)	3405 (29.2)	0.038
Vedolizumab	2 (0.1)	41 (2.5)	
Ustekinumab	-	19 (0.16)	
Others	2 (0.1)	4 (0.03)	
IBD-related surgery	389/1763 (22.1)	2742/11651 (23.5)	0.184
Abdominal surgery for CD^*^	276 (29.4)	2253 (34.5)	0.002
Total proctocolectomy for UC^**^	37 (3.5)	240 (3.4)	1
Perianal surgery	154 (8.3)	1372 (11.8)	0.184

*Percentages are calculated based on patients with available data*.

* *Percentage calculated on CD patients*.

** *Percentage calculated on UC patients*.

**Figure 4:**
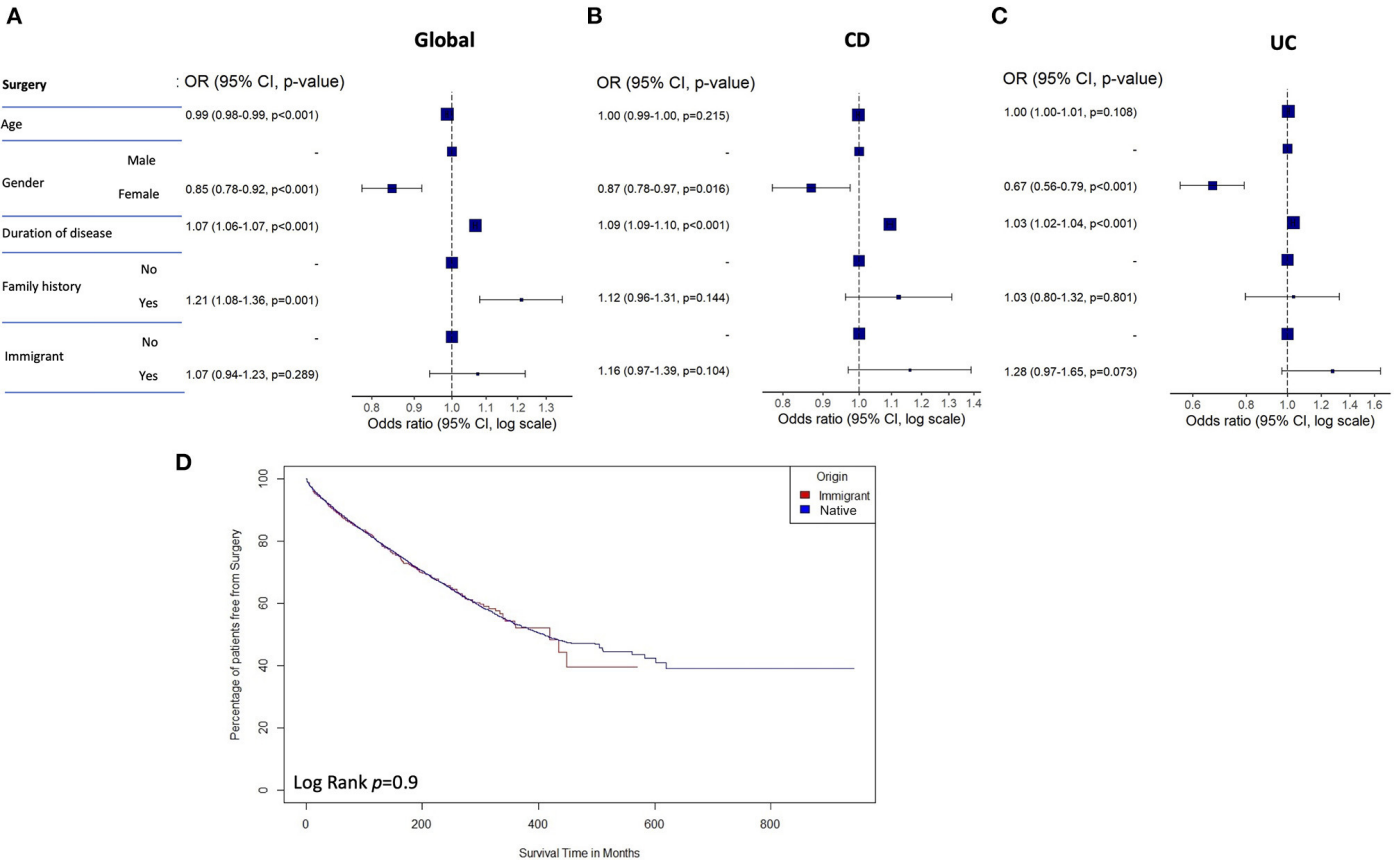
Forest plots representing the odds ratio of surgery in the multivariate analysis for the global **(A)**, CD **(B)**, and UC **(C)** cohorts of patients. **(D)** Survival analysis of the time to surgery in immigrant vs. native patients. CD, Crohn's disease; UC, ulcerative colitis.

No significant differences were found in the frequency of IBD-related abdominal and perianal surgeries between all immigrant and native IBD patients, or in the frequency of proctocolectomy between native and immigrant UC patients. However, abdominal surgery was significantly more frequent among native vs. immigrant CD patients. The risk of surgical treatment was not associated with immigrant status. Female sex, longer duration of disease, and family history of IBD were associated with this parameter in the global cohort of all IBD patients (Figure 4A), although only female sex and longer duration of disease remained independently associated, with a significantly decreased risk of surgical treatment, when CD and UC cohorts of patients were analyzed separately (Figures 4B,C). Supplementary Table 9 shows the univariate and multivariate analyses for the risk for surgical treatment in the series of IBD patients. Time to surgery during follow-up was not different between native vs. immigrant patients according to the performed survival analysis of the time to intestinal surgery (Figure 4D).

Loss to follow-up was more frequent among immigrant IBD patients (*n* = 265, 14.6 vs. *n* = 1,216, 10.4%; *p* = 0.001). Mean follow-up was 5.6 ± 6.2 years in the immigrant subgroup and 6.1 ± 4.5 years in the subgroup of native patients (*p* = 0.016). Significantly more native than immigrant patients died during the study period (2.6%, *n* = 300 vs. 0.9%, *n* = 17; *p* < 0.01). The cause of death was available for 179 natives (59.6%) and 3 immigrants (17.6%). The most frequent causes of death in native patients were neoplasm (*n* = 97, 54.2%) and infection (*n* = 30, 16.7%). The three recorded causes of death among immigrants were neoplasm (*n* = 1), infection (*n* = 1), and others (*n* = 1). Supplementary Table 10 shows the recorded causes of death in both subgroups.

## Discussion

We present the first study exploring the characteristics of first-generation immigrant IBD population in Spain, extracted from the vast ENEIDA nationwide registry of GETECCU. The immigrant cohort (>1,800 patients) was younger at disease onset and showed an increased risk of having EIMs and using biologics compared with the native cohort (>11,000 patients). Our results add to the scarce epidemiologic data characterizing immigrant IBD patients in Southern Europe, and suggest that immigrant IBD patients in Spain may have a distinct IBD phenotype.

The population of first-generation immigrants diagnosed with IBD and living in Spain is mainly Caucasian and Latin American, though a large proportion of patients are of Arab ethnicity. Asian patients make up a far smaller section of the population, unlike in series evaluated in other studies—mainly from the USA and the UK—in which South Asians constitute the most prevalent ethnic group (19–21). While those same studies found no differences in age at disease onset, we present data showing that the first-generation-immigrant IBD population in Spain is younger than native patients at disease onset and has a shorter disease duration. To explain the observed difference in age at onset, we may speculate on the relevance of the so called exposome (22), or the accumulation of environmental exposures, including several disease triggering factors such as microbiota dysbiosis, smoking and use of antibiotics. A study by Damas et al. (23) found earlier IBD onset among young Cuban migrants to America compared with previous migratory cohorts, suggesting a more intensive exposure to environmental triggering factors in the more recent arrivals. We also found that patients who were younger than 15 years on arrival in Spain were significantly younger at disease onset than those who migrated after 15. This fact highlights the relevance of early-life exposure to environmental risk factors for disease. Age of migration is an important factor in IBD development and phenotype, with younger ages being more affected in terms of incidence. In line with observations from our study, South Asians younger than 5 years-old at migration to US, or descendants who were already born in US, were younger at IBD onset than those who were older than 5 years-old at migration (24).

UC predominance over CD in IBD diagnosis has been described in certain ethnic groups such as South Asian and Hispanic (25–27). Our study also found a higher proportion of UC among Latin American and Asian patients compared with other ethnicities. This finding is consistent with findings reported of South Asians cohorts in the UK and North America, where the burden of UC is believed to be twice that of CD (19, 21, 24). Indeed, one meta-analysis showed that UC was more frequent among Hispanic vs. non-Hispanic Americans (28), and studies conducted in Mexico and South America have reported UC to be the predominant IBD subtype (29–32). Interestingly, other series have found that Hispanic patients are not only more likely to have UC but are also less likely than the non-Hispanic white population to have a family history of IBD (33, 34). We also found a lower frequency of family history of the disease among our immigrant cohort, which suggests that the cause is more likely to be de novo or sporadic. This observation is in line with other reports of sporadic disease among non-Caucasian ethnic groups (35, 36). On the other hand, several studies have reported the inverse association between smoking and presenting UC (37), which may also help to explain the predominance of UC in Latin American and Asian patients, who are less likely to smoke than Caucasian immigrants and native patients.

Several studies have reported a more aggressive phenotype, defined as more frequently colonic and perianal, and with more penetrating and stricturing behavior, in South Asian migrants (19, 20, 24). We did not observe differences in either CD location or UC extent in our study, possibly because Asian patients represented only a small part of the immigrant series. We did find more frequent stricturing behavior among native vs. immigrant CD patients, although stricturing was only associated with longer disease duration in the multivariate analysis. The longer disease history probably reflects the accumulation of disease progression milestones. Similar results were obtained in a multicenter study on African-American compared with Caucasian patients (38). Although other studies showed no differences in behavior between immigrant and host populations (20), one potential explanation for increased stricturing in our native cohort may derive from the genetic perspective. The allele frequency of various mutations within NOD2/ CARD15 may help to explain differences in ethnic risks (39). Indeed, NOD2 variations are less frequent among African Americans (40), and South Asian patients compared with Caucasian patients (41, 42). A second explanation may relate to the higher prevalence of smoking habit among native vs. immigrant patients. Smoking has been widely described as a risk factor for developing CD, determining disease location and the development of strictures or fistulae, and increasing the risk of surgery (43–45).

The two main differences between first-generation immigrant and native IBD populations in Spain were the increased risk of EIMs and use of biologics among immigrants. Previous studies on immigrant populations have not reported these findings (19, 21, 24). In fact, a lower rate of EIMs has previously been described in Asian patients (46, 47). It is possible that we found the opposite association because our cohort contained relatively few Asian patients, and indeed, our sub-analysis of the different ethnic groups showed increased rates of EIMs in Caucasian, Latin American and Arab patients, but not in Asian patients. Although the physiological mechanisms of EIM development are poorly understood, several risks factors have been described, including longer disease duration, colonic CD, and female sex (48, 49). In our study, longer disease duration, female sex and immigrant status were risk factors independently associated with EIM development. Increased risk of EIMs can therefore be considered a component of the IBD phenotype in our immigrant population.

The use of biologics, meanwhile, was independently associated with longer disease duration, younger age, and immigrant status. The increased frequency of EIMs among immigrants may explain this result, as anti-TNF is one of the most effective treatments for managing EIMs in IBD (49, 50). In addition, younger disease onset, as found in our immigrant cohort, is a poor prognostic factor (51), which may also help to explain the increased use of biologics among our immigrant population. Interestingly, time to first biologic treatment during follow-up was shorter in the immigrant population. Economic factors are thought to be the primary driver of racial and ethnic disparities in health services utilization, including use of prescription medication (52). However, our results show that both native and immigrant populations have full access to biologic therapies under the universal healthcare coverage provided by Spain's public health service, and low socioeconomic status is therefore unlikely to have contributed to differences in prescription patterns in this study. On the other hand, clinical practice among ENEIDA researchers is fairly uniform due to Spanish program of certification of quality of care at inflammatory bowel disease units (53), which sets the standards of care in IBD all over the country. An alternative explanation is that immigrant patients may have a distinctive disease course. No differences in the risk of IBD-related surgery were found between first-generation-immigrant and native IBD populations in Spain. However, intestinal resection was more frequent among native CD patients. This may be related to the increased rate of stricturing behavior and smoking habit, both described as risk factors for surgery in CD (44).

We must acknowledge that the retrospective study design and the lack of information regarding patient's genetic background, dietary habits and socioeconomical status constitute limitations of the present study. On the other hand, the main strengths of our study were the large cohorts; the representation of several ethnicities, including poorly studied groups such as Latin American or Arab immigrants; and the inclusion of patients diagnosed before 2015 to guarantee a follow-up period of at least 5 years.

In summary, we present the first study exploring the characteristics of first-generation immigrants with IBD in Spain.Immigrant patients were younger at disease onset and showed an increased risk of having EIMs and using biologics compared with native patients. These factors could constitute features of an IBD phenotype for the immigrant population in Spain. Our study represents a new landmark in the epidemiologic characterization of IBD immigrant patients in Southern Europe.

## Data Availability Statement

The original contributions presented in the study are included in the article/Supplementary Material, further inquiries can be directed to the corresponding authors.

## Ethics Statement

The studies involving human participants were reviewed and approved by Ethics Committee from Hospital General Universitario de Alicante. The patients/participants provided their written informed consent to participate in this study.

## Author Contributions

AG and RF: planning and/or conducting the study. AG, ER, MG-V, JG, DO, IV, MM, JPG, MA, ES-R, MB-W, VL, BC, IM-J, YZ, MM-A, RM, MN, ESi, LM, MVe, JP-C, ESa, XC, LA, VM, FB, LF-S, MVa, LD, CR, CM-V, RL, MRi, EI, BH, DB, JR, MM-M, MRo, OR, EH, MS, JB, RD, JH, OM, DC, DG, FM, MP, PA, FA-A, GA, LB, NM, AL, PV, IR-L, LR, LS, ESe, MB-d, and ED: collecting data. AG, RF, and PZ: interpreting data and drafting of the manuscript. All authors contributed to the article and approved the submitted version.

## Funding

The ENEIDA registry of GETECCU is supported by AbbVie, Galápagos, Janssen, Biogen, Takeda, Pfizer. AG is recipient of a grant by Instituto de Salud Carlos III, Madrid, Spain (PI21/1702).

## Conflict of Interest

AG has served as speaker, consultant and advisory member for or has received research funding from MSD, Abbvie, Pfizer, Kern Pharma, Takeda, Janssen, Ferring, Faes Farma, Shire Pharmaceuticals, Tillotts Pharma, Chiesi and Otsuka Pharmaceutical. ER has provided scientific advice/participated in medical meetings/received research funding from/received payment for presentations and advice from: MSD, Schering-Plow, Ferring, Abbvie, Takeda, Janssen, Fresenius Kabi, Pfizer. IV has served as a speaker, or has received research or education funding from Abbvie, MSD, Pfizer, Takeda, Janssen, Tillotts Pharma, Ferring and Shire Pharmaceuticals. MM has served as speaker or has received research funding from MSD, Abbvie, Pfizer, Kern Pharma, Takeda, Janssen, Ferring, Faes Farma, Shire Pharmaceuticals, Dr. Falk Pharma, Biogen, Tillotts Pharma, Chiesi and Adacyte. JPG has served as speaker, consultant, and advisory member for or has received research funding from MSD, Abbvie, Pfizer, Kern Pharma, Biogen, Mylan, Takeda, Janssen, Roche, Sandoz, Celgene, Gilead/Galapagos, Ferring, Faes Farma, Shire Pharmaceuticals, Dr. Falk Pharma, Tillotts Pharma, Chiesi, Casen Fleet, Gebro Pharma, Otsuka Pharmaceutical, and Vifor Pharma. YZ has received support for conference attendance, speaker fees, research support and consulting fees from Abbvie, Adacyte, Almirall, Amgen, Dr. Falk, FAES Pharma, Ferring, Janssen, MSD, Otsuka, Pfizer, Shire, Takeda and Tillots. XC has received grants for research from Abbvie, MSD, Vifor fees for advisory boards form Abbvie, MSD, Takeda, Pfizer, Janssen and VIFOR and has given lectures for Abbvie, MSD, Janssen, Pfizer, Takeda, Shire and Allergan. FB has served as a speaker, a consultant and advisory member for or has received research funding from MSD, Abbvie, Takeda, Janssen, Pfizer, Biogen, Amgen, Ferring, Faes Farma, Tillotts Pharma, Falk Pharma, Chiesi, Gebro Pharma, Vifor Pharma. MM-M has served as speaker, consultant and advisory member for or has received research funding from MSD, Abbvie, Pfizer, Kern Pharma, Takeda, Janssen, Shire Pharmaceuticals and Otsuka Pharmaceutical. MR has served as speaker, consultant or advisory member for MSD, Abbvie, Pfizer, Takeda, Janssen, Ferring and Chiesi. MP has served as speaker or has received research funding from Abbvie, Takeda and Janssen. IR-L has received financial support for traveling and educational activities from or has served as an advisory board member for MSD, Pfizer, Abbvie, Takeda, Janssen, Tillotts Pharma, Shire Pharmaceuticals, Roche, Celltrion, Faes Farma, Ferring, Dr. Falk Pharma, Otsuka Pharmaceutical and Adacyte. Financial support for research from Tillotts Pharma. RL has served as a speaker, or has received research or education funding from MSD, Abbvie, Pfizer, Takeda, Janssen and Dr. Falk. LA has served as speaker, or has received research or education funding from MSD, Abbvie, Kern Pharma, Ferring, FaesFarma, Shire Pharmaceuticals, Pfizer, Takeda, Janssen, Tillotts Pharma, and Otsuka Pharmaceutical. FA-A has served as speaker, consultant and advisory member for or has received research funding from MSD, Abbvie, Pfizer, Kern Pharma, Takeda, Janssen, Ferring, Faes Farma, Shire Pharmaceuticals, Tillotts Pharma, Chiesi and Dr. Falk. LR has served as a speaker, or has received education funding from MSD, Abbvie, Adacyte, Takeda, Pfizer, Janssen and Ferring. MB-d has served as a speaker, consultant and advisory member for or has received research funding from MSD, AbbVie, Janssen, Kern Pharma, Celltrion, Takeda, Gillead, Celgene, Pfizer, Sandoz, Biogen, Fresenius, Ferring, Faes Farma, Dr. Falk Pharma, Chiesi, Gebro Pharma, Adacyte and Vifor Pharma. ED has served as a speaker, or has received research or education funding or advisory fees from AbbVie, Adacyte Therapeutics, Biogen, Celltrion, Gilead, Janssen, Kern Pharma, MSD, Pfizer, Roche, Samsung, Takeda, Tillots, Thermofisher. RF has served as a speaker, or has received research or education funding or advisory fees from AbbVie, Janssen, Takeda, Adacyte Therapeutics, Sanofi, GlaxoSmithKline, Almirall. The remaining authors declare that the research was conducted in the absence of any commercial or financial relationships that could be construed as a potential conflict of interest.

## Publisher's Note

All claims expressed in this article are solely those of the authors and do not necessarily represent those of their affiliated organizations, or those of the publisher, the editors and the reviewers. Any product that may be evaluated in this article, or claim that may be made by its manufacturer, is not guaranteed or endorsed by the publisher.
