# Supplementary material for: Immigrant IBD Patients in Spain Are Younger, Have More Extraintestinal Manifestations and Use More Biologics Than Native Patients
Source: Front Med (Lausanne). 2022 Feb 1;9:823900. doi: 10.3389/fmed.2022.823900 (PMC8844561; doi:10.3389/fmed.2022.823900)
Supplement: Supplementary file 1 [file Data_Sheet_1.docx]

**Supplementary Table 1.** Main demographic characteristics of immigrants and native CD patients.

|  | Immigrants | natives | p |
| --- | --- | --- | --- |
|  |  |  |  |
| Gender, female (n,%) | 373 (48.1) | 2302 (48.4) | 0.889 |
| Mean current age (SD) | 44.1 (12.6) | 51.8 (15.2) | <0.001 |
| Mean age at IBD diagnosis | 29.7 (12.2) | 33.1 (14.9) | <0.001 |
| Mean duration of disease (SD) | 14.5 (7.7) | 18.7 (8.4) | <0.001 |
| Smoking habit |  |  |  |
| no (n,%) | 430 (60.2) | 2278 (47.8) | <0.001 |
| yes (n,%) | 179 (25.1) | 1423 (29.9) |  |
| Ex (n,%) | 105 (14.7) | 1063 (22.3) |  |
| Crohn´s age at diagnosis |  |  |  |
| A1 (<16y) (n,%) | 67 (8.6) | 275 (5.8) | <0.001 |
| A2(16-40y) (n,%) | 586 (75.3) | 3223 (67.8) |  |
| A3>40y (n,%) | 125 (16.1) | 1257 (26.4) |  |
| Crohn´s location |  |  |  |
| L1(ileal) (n,%) | 154 (28.5) | 1025 (28.4) | 0.368 |
| L2(colonic) (n,%) | 111 (20.6) | 640 (17.7) |  |
| L3(ileocolonic) (n,%) | 233 (43.1) | 1636 (45.3) |  |
| L4(upper GI) (n,%) | 42 (7.8) | 308 (8.5) |  |
| Crohn´s behavior |  |  |  |
| B1(inflammatory) | 482 (63.9) | 2744 (57.7) | 0.002 |
| B2(stricturing) (n,%) | 144 (19.1) | 1158 (24.4) |  |
| B3(perforating) (n,%) | 128 (17.0) | 853 (17.9) |  |
| Perianal perforating (n;%) | 242 (13.7) | 1776 (15.4) | 0.069 |
| Extraintestinal manifestations (n;%) | 159 (24.1) | 753 (15.8) | <0.001 |
| Family history of IBD (n,%) | 71 (10.4) | 796 (16.7) | <0.001 |

**Supplementary Table 2.** Main demographic characteristics of immigrants and native UC patients.

|  | Immigrants | natives | p |
| --- | --- | --- | --- |
|  |  |  |  |
| Gender, female (n,%) | 536 (52.1) | 3031 (45.5) | <0.001 |
| Mean current age (SD) | 46.1 (12.6) | 56.4 (16.2) | <0.001 |
| Mean age at IBD diagnosis | 32.3 (11.6) | 38.8 (15.6) | <0.001 |
| Mean duration of disease (SD) | 13.8 (6.8) | 17.5 (8.9) | <0.001 |
| Smoking habit |  |  |  |
| no (n,%) | 716 (76.2) | 4509 (67.5) | <0.001 |
| yes (n,%) | 108 (11.5) | 767 (11.5) |  |
| Ex (n,%) | 116 (12.3) | 1407 (21.1) |  |
| Ulcerative colitis extent |  |  |  |
| Proctitis (n,%) | 186 (29.7) | 1478 (31.9) | 0.031 |
| Left sided colitis (n,%) | 357 (57.0) | 2689 (58.1) |  |
| Extensive colitis (n,%) | 83 (13.3) | 459 (9.9) |  |
| Extraintestinal manifestations (n;%) | 148 (15.3) | 494 (7.4) | <0.001 |
| Family history of IBD (n,%) | 83 (9) | 852 (12.7) | 0.001 |

**Supplementary Table 3.** Main demographic and clinical characteristics of immigrants under and above 15 years old at migration.

|  | Immigrants younger than 15 | Immigrants older than 15 | p |
| --- | --- | --- | --- |
| n | 181 | 1048 |  |
| Gender/female(%) | 90(50) | 520(49.6) | 0.989 |
| Age, mean(SD) | 36.31 (13.80) | 45.63 (11.53) | <0.001 |
| Age of onset, mean(SD) | 23.24 (11.34) | 32.12 (11.28) | <0.001 |
| Age at migration, mean(SD) | 7.76 (4.39) | 29.84 (9.77) | <0.001 |
| Disease duration, mean(SD) | 13.07 (6.84) | 13.48 (6.91) | 0.465 |
| Smoking habit, n(%) |  |  | 0.186 |
| Ex | 16 (9.5) | 131 (13.4) |  |
| No | 117 (69.6) | 685 (70.2) |  |
| Yes | 35 (20.8) | 160 (16.4) |  |
| IBD |  |  | 0.02 |
| Ulcerative colitis (n,%) | 89 (49.2) | 613 (58.8) |  |
| IBDU (n,%) | 2 (1.1) | 23 (2.2) |  |
| Crohn´s disease(n,%) | 90 (49.7) | 406 (39.0) |  |
| Crohn´s age at diagnosis |  |  | <0.001 |
| A1 (<16y) (n,%) | 42 (23.2) | 36 (3.5) |  |
| A2(16-40y)(n,%) | 125 (69.1) | 798 (76.5) |  |
| A3>40y(n,%) | 14 (7.7) | 209 (20.0) |  |
| Crohn´s location |  |  | 0.773 |
| L1(ileal) (n,%) | 19 (20.7) | 89 (20.0) |  |
| L2(colonic) (n,%) | 41 (44.6) | 208 (46.8) |  |
| L3(ileocolonic) (n,%) | 25 (27.2) | 125 (28.2) |  |
| L4(upper GI) (n,%) | 7 (7.6) | 22 (5.0) |  |
| Crohn´s behavior |  |  | 0.214 |
| B1(inflammatory) | 57 (67.1) | 273 (65.9) |  |
| B2(stricturing) (n,%) | 19 (22.4) | 70 (16.9) |  |
| B3(perforating) (n,%) | 9 (10.6) | 71 (17.1) |  |
| Perianal perforating (n;%) | 32 (18.0) | 142 (13.8) | 0.227 |
| Ulcerative colitis extent |  |  | 0.308 |
| Proctitis (n,%) | 4 (7.3) | 54 (14.9) |  |
| Left sided colitis (n,%) | 17 (30.9) | 98 (26.9) |  |
| Extensive colitis (n,%) | 34 (61.8) | 212 (58.2) |  |
| Extraintestinal manifestations (n;%) | 36 (23.3) | 180 (18.9) | 0.237 |
| Family history of IBD (n,%) | 22 (14.1) | 87 (9.2) | 0.082 |

**Supplementary Table 4.** Main demographic characteristics of immigrants diagnosed in Spain *versus* outside Spain.

|  |  | Immigrants | | |  |  |
| --- | --- | --- | --- | --- | --- | --- |
|  | Natives (N=11687) 1 | Diagnosed in Spain (N=1043) 2 | Diagnosed outside Spain (N=415) 3 | Total (N=1458) | p value (1 vs 2) | p value (2 vs 3) |
| Age |  |  |  |  | < 0.001 | < 0.001 |
| Mean (SD) | 54.477 (15.972) | 45.402 (12.400) | 42.619 (11.588) | 44.609 (12.235) |  |  |
| Range | 16.000 - 120.000 | 18.000 - 93.000 | 17.000 - 83.000 | 17.000 - 93.000 |  |  |
| Gender |  |  |  |  | 0.102 | 0.491 |
| Male | 6230 (53.3%) | 528 (50.7%) | 202 (48.7%) | 730 (50.1%) |  |  |
| Female | 5457 (46.7%) | 514 (49.3%) | 213 (51.3%) | 727 (49.9%) |  |  |
| Age of onset |  |  |  |  | < 0.001 | < 0.001 |
| Mean (SD) | 36.520 (15.602) | 33.391 (11.810) | 24.945 (9.373) | 30.985 (11.800) |  |  |
| Range | 0.000 - 104.000 | 8.000 - 83.000 | 4.000 - 67.000 | 4.000 - 83.000 |  |  |
| Disease duration |  |  |  |  | < 0.001 | < 0.001 |
| Mean (SD) | 17.957 (8.732) | 12.024 (5.614) | 17.675 (8.167) | 13.632 (6.929) |  |  |
| Range | 6.000 - 78.000 | 6.000 - 44.000 | 6.000 - 47.000 | 6.000 - 47.000 |  |  |
| Smoking habit |  |  |  |  | < 0.001 | 0.331 |
| Ex | 2508 (21.5%) | 135 (13.8%) | 41 (10.9%) | 176 (13.0%) |  |  |
| No | 6951 (59.5%) | 683 (69.8%) | 266 (70.9%) | 949 (70.1%) |  |  |
| Yes | 2227 (19.1%) | 161 (16.4%) | 68 (18.1%) | 229 (16.9%) |  |  |
| IBD |  |  |  |  | 0.052 | 0.007 |
| Ulcerative colitis | 6682 (57.2%) | 618 (59.3%) | 211 (50.8%) | 829 (56.9%) |  |  |
| IBDU | 240 (2.1%) | 30 (2.9%) | 10 (2.4%) | 40 (2.7%) |  |  |
| Crohn's disease | 4765 (40.8%) | 394 (37.8%) | 194 (46.7%) | 588 (40.4%) |  |  |
| Family history |  |  |  |  | < 0.001 | 0.114 |
| No | 10010 (85.7%) | 849 (91.0%) | 326 (88.1%) | 1175 (90.2%) |  |  |
| Yes | 1676 (14.3%) | 84 (9.0%) | 44 (11.9%) | 128 (9.8%) |  |  |
| EIMs |  |  |  |  | < 0.001 | 0.625 |
| No | 10411 (89.1%) | 767 (80.2%) | 298 (81.4%) | 1065 (80.6%) |  |  |
| Yes | 1275 (10.9%) | 189 (19.8%) | 68 (18.6%) | 257 (19.4%) |  |  |

**Supplementary Table 5.** Main characteristics of immigrant patients by ethnicity.

|  | Caucasian | Latin American | Arabian | Asian | p |
| --- | --- | --- | --- | --- | --- |
|  | (n=771) | (n=572) | (n=341) | (n=125) |  |
| Gender, female (n,%) | 414 (53.8) | 338 (59.1) | 120 (35.2) | 34 (27,2) | <0.001 |
| Mean current age (SD) | 43.9 (12.1) | 46.3 (12.9) | 46.6 (12.8) | 44.7 (12.6) | 0.001 |
| Mean age at IBD diagnosis | 28.7 (11.3) | 33.0 (12.1) | 33.3 (12.4) | 32.2 (11.0) | <0.001 |
| Mean duration of disease (SD) | 15.2 (7.9) | 13.4 (6.6) | 13.3 (6.5) | 12.5 (5.4) | <0.001 |
| Smoking habit |  |  |  |  |  |
| no (n,%) | 429 (61.1) | 388 (74.2) | 234 (74.1) | 94 (81.7) | <0.001 |
| yes (n,%) | 166 (23.6) | 63 (12.0) | 40 (12.7) | 16 (13.9) |  |
| ex(n,%) | 107 (15.2) | 72 (13.8) | 42 (13.3) | 5 (4.3) |  |
| Crohn´s disease (n,%) | 368 (48.0) | 182 (31.9) | 170 (49.9) | 33 (26.4) | <0.001 |
| Ulcerative colitis (n,%) | 384 (50.1) | 364 (63.9) | 165 (48.4) | 86 (68.8) |  |
| Unclassified IBD (n,%) | 15 (2.0) | 24 (4.2) | 6 (1.8) | 6 (4.8) |  |
| Crohn´s age at diagnosis |  |  |  |  |  |
| A1 (<16y) (n,%) | 43 (11.7) | 8 (4.4) | 13 (7.6) | 1 (3.0) | 0.001 |
| A2(16-40y)(n,%) | 285 (77.4) | 134 (73.6) | 123 (72.4) | 26 (78.8) |  |
| A3>40y(n,%) | 40 (10.9) | 40 (22.0) | 34 (20.0) | 6 (18.2) |  |
| Crohn´s location |  |  |  |  |  |
| L1(ileal) (n,%) | 74 (29.4) | 35 (27.8) | 32 (25.38) | 6 (27.3) | 0.329 |
| L2(colonic) (n,%) | 51 (20.2) | 34 (27.0) | 17 (13.7) | 5 (22.7) |  |
| L3(ileocolonic) (n,%) | 105 (41.7) | 48 (38.1) | 66 (53.2) | 10 (45.5) |  |
| L4(upper GI) (n,%) | 22 (8.7) | 9 (7.1) | 9 (7.3) | 1 (4.5) |  |
| Crohn´s behaviour |  |  |  |  |  |
| B1(inflammatory) | 231 (64.9) | 119 (66.9) | 95 (57.9) | 25 (75.8) | 0.282 |
| B2(stricturing) (n,%) | 70 (19.7) | 27 (15.2) | 36 (22.0) | 3 (9.1) |  |
| B3(perforating) (n,%) | 55 (15.4) | 32 (18.0) | 33 (20.1) | 5 (15.2) |  |
| Perianal perforating (n;%) | 111 (15.1) | 59 (10.8) | 51 (15.8) | 13 (11.7) | 0.078 |
| Ulcerative colitis extent |  |  |  |  |  |
| Proctitis (n,%) | 33 (14.2) | 31 (14.7) | 11 (9.9) | 6 (11.8) | 0.812 |
| Left sided colitis (n,%) | 73 (31.3) | 59 (28.0) | 34 (30.6) | 15 (29.4) |  |
| Extensive colitis (n,%) | 127 (54.5) | 121 (57.3) | 66 (59.5) | 30 (58.8) |  |
| Extraintestinal manifestations (n;%) | 133 (18.7) | 109 (20.4) | 59 (18.8) | 10 (8.6) | 0.032 |
| Family history of IBD (n,%) | 77 (11.3) | 34 (6.6) | 32 (10.5) | 7 (6.4) | 0.024 |
| Endoscopic treatment | 13 (2.1) | 5 (1.1) | 3 (1.0) | 2 (2.0) | 0.504 |
| Immunosuppresants | 449 (59.2) | 259 (45.9) | 207 (61.4) | 61 (48.8) | <0.001 |
| Biologics* | 306 (41.6) | 168 (31.2) | 118 (36.4) | 38 (32.2) | 0.001 |
| AntiTNF (adalimumab) | 204 (36.4) | 101 (30.2) | 54 (28.0) | 10 (15.2) | <0.001 |
| AntiTNF (infliximab) | 286 (51.1) | 159 (47.6) | 118 (61.1) | 46 (69.7) |  |
| AntiTNF (others) | 16 (2.9) | 18 (5.4) | 7 (3.6) | 2 (3.0) |  |
| Others | 6 (1.1) | 2 (0.6) | 0 (0.0) | 0 (0.0) |  |
| Ustekinumab | 20 (3.6) | 20 (6.0) | 8 (4.1) | 2 (3.0) |  |
| Vedolizumab | 28 (5.0) | 34 (10.2) | 6 (3.1) | 6 (9.1) |  |
| IBD-related surgery | 187 (25.6) | 83 (15.5) | 95 (28.9) | 12 (10.1) | <0.001 |
| Abdominal surgery for CD | 130 (28.8) | 57 (25.6) | 68 (35.1) | 8 (21.1) | 0.117 |
| Total proctocolectomy for UC | 30 (7.6) | 19 (5.0) | 12 (6.9) | 2 (2.2) | 0.185 |
| Perianal surgery | 72 (9.3) | 38 (6.6) | 31 (9.1) | 3 (2.4) | 0.114 |
| * Several patients had been treated with more than one biologic. | | |  |  |  |

**Supplementary Table 6.** Univariate and multivariate analyses for structuring behavior in CD patients.

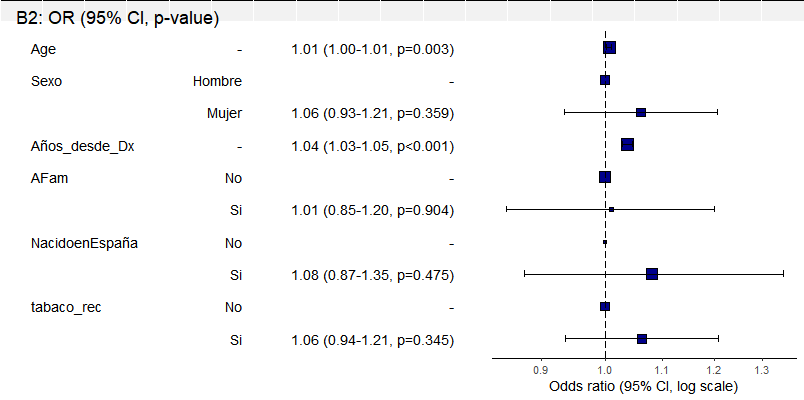


Age

Gender

Male

Female

Disease duration

Yes

Born in Spain

No

No

Yes

Family history

**Stricturing behavior**

Smoking habit

Yes

No

**Supplementary Table 7.** Univariate and multivariate analyses showing the odds ratio for EIMs in the global cohort of IBD patients, and CD and UC subpopulations.

**Supplementary Table 8.** Univariate and multivariate analyses showing the odds ratio for biological treatment in the global cohort of IBD patients, and CD and UC subpopulations.

**Supplementary Table 9.** Univariate and multivariate analyses showing the odds ratio for surgery in the global cohort of IBD patients, and CD and UC subpopulations.

**Supplementary Table 10.** Available causes of death among immigrants and native IBD patients.

|  | Immigrants | Natives |
| --- | --- | --- |
| Cause (N) | (n=17) | (n=300) |
| Cancer | 1 | 97 |
| Infection | 1 | 30 |
| Cardiovascular diseases |  | 13 |
| Cerebrovascular diseases |  | 10 |
| Chronic obstructive pulmonay diseases |  | 11 |
| Other | 1 | 18 |
| Not collected in ENEIDA registry | 14 | 121 |
